# Effect of Myclobutanil Pesticide on the Physiological Behavior of Two Newly Isolated *Saccharomyces cerevisiae* Strains during Very-High-Gravity Alcoholic Fermentation

**DOI:** 10.3390/microorganisms7120666

**Published:** 2019-12-09

**Authors:** Antonia Terpou, Maria Dimopoulou, Aikaterini Belka, Stamatina Kallithraka, George-John E. Nychas, Seraphim Papanikolaou

**Affiliations:** Department of Food Science and Human Nutrition, Agricultural University of Athens, 75 Iera Odos, 11855 Athens, Greece; aterpou@upatras.gr (A.T.); marouladimo@aua.gr (M.D.); katerina_be@hotmail.com (A.B.); stamatina@aua.gr (S.K.); gjn@aua.gr (G.-J.E.N.)

**Keywords:** *Saccharomyces cerevisiae*, ethanol production, very-high-gravity fermentation, myclobutanil

## Abstract

Yeasts are able to act as biosorbents, as their cell wall includes several components capable of binding organic xenobiotic compounds that can potentially be removed during various fermentation processes. In the present investigation, two novel *Saccharomyces cerevisiae* strains (LMBF-Y 16 and LMBF-Y-18), previously isolated from grapes, were studied regarding their physiological behavior (dry cell weight—DCW production, substrate uptake, and ethanol and glycerol biosynthesis) during fermentations of grape must, in some cases enriched with commercial glucose and fructose (initial total sugar concentration approximately 150 and 250 g/L, respectively). Myclobutanil (a chiral triazole fungicide broadly used as a protective agent of vine) was also added to the culture media at various concentrations in order to assess the ability of the yeasts to simultaneously perform alcoholic fermentations and detoxify the medium (i.e., to remove the fungicide). In the first set of experiments and for both tested strains, trials were carried out in either 250 mL or 2.0 L agitated shake flasks in either synthetic glucose-based experiments or grape musts. Since the results obtained in the trials where the cultures were placed in 2.0 L flasks with grape musts as substrates were superior in terms of both DCW and ethanol production, these experimental conditions were selected for the subsequent studies. Both strains showed high fermentative efficiency, producing high amounts of DCW (9.5–10.5 g/L) in parallel with high ethanol production, which in some cases achieved values very close to the maximum theoretical ethanol production yield (≈0.49 g of ethanol per g of sugar). When using grape must with initial total sugars at approximately 250 g/L (very high gravity fermentation media, close to winemaking conditions), significantly high ethanol quantities (i.e., ranging between 105 and 123 g/L) were produced. Myclobutanil addition slightly negatively affected sugar conversion into ethanol; however, in all cases, ethanol production was very satisfactory. A non-negligible myclobutanil removal during fermentation, which ranged between 5%–27%, as a result of the adsorptive or degradative capacity of the yeast was also reported. The presence of myclobutanil had no effect on DCW production and resulted in no significant differences in the biosynthesis of glycerol. Therefore, these newly isolated yeast strains could be excellent candidates for simultaneous high ethanol production and parallel pesticide removal in a general biorefinery concept demonstrating many environmental benefits.

## 1. Introduction

A significant volume of research in industrial biotechnology focuses on the isolation of or genetic manipulation for the construction of novel robust, high-performing microorganisms useful for the production of improved products presenting technological interest [1]. In this context, many recent scientific studies have been conducted targeting the discovery of new strains for the optimization of ethanol production, mainly aiming to achieve high yields, high final product concentrations, and high volumetric productivities [2,3]. In fact, research into the production of bioethanol falls mainly on two axes: the first axis, “systems biology”, is related to the construction of mutant or genetically engineered strains capable of producing ethanol at significant final product concentrations and high volumetric productivities and/or producing, in small quantities, metabolites antagonistic to ethanol (i.e., glycerol). Therefore, several genetic engineering studies have been carried out in order to construct strains (mainly of the microorganisms *Saccharomyces cerevisiae* and *Zymomonas mobilis*) capable of consuming pentoses (e.g., xylose, arabinose) and degrading macromolecules (e.g., starch, cellulose, etc.) while incapable of producing compounds antagonistic to sugar → ethanol pathway (like glycerol) [4,5,6,7]. On the second axis, research activities concerning bioethanol production have focused on the process optimization, bioprocess modeling, and the development of novel fermentation strategies and configurations (e.g., simultaneous saccharification and fermentation, consolidated bioprocess, etc.) [4,5,8,9,10]. To this end, the establishment of fermentation when very high initial quantities of sugars (i.e., ≥250 g/L) are employed as substrate (so-called “very-high-gravity fermentation”), as well as the accomplishment of fermentation under non-aseptic conditions, has recently gained significant attention [5,11,12]. Both these strategies may offer great savings in process water and energy requirements, and when appropriate microbial strains are found for these purposes, no major alterations to the production lines of existing bioethanol production plants are required [11,13].

Among other microorganisms, yeasts have a rich history as well as a bright future in the industrial biotechnology. Specifically, yeasts play an essential role in ethanol production by fermenting a wide range of sugars and achieving high yields and productivities [14]. Historically, indigenous yeast strains naturally occurring on grapes or/and in winemaking environments have been responsible for the fermentation of grape juice [15]. Specifically, during spontaneous fermentation of grape juice, indigenous yeast strains belonging to the genera *Hanseniaspora*, *Candida, Kloeckera*, and *Pichia* may be detected in the early stages of fermentation, while ethanol-tolerant *S. cerevisiae* species are mainly detected in the middle and final stages of fermentation [16]. Nowadays, wine production is achieved by inoculation with selected yeast starter strains selected for their enological properties [17,18,19]. However, unidentified yeast strains may provide many biotechnological advances and can be recovered the through isolation of novel strains recovered from “wild” grape microflora [20,21].

The grape microbial ecosystem is composed of highly diverse microorganisms, including yeasts, bacteria, and fungi. Yeasts and bacteria are regarded as the principal agents of wine fermentation, but there is also interest in other microorganisms considered to act as spoilage agents. These agents include filamentous fungi, which may greatly influence the safety characteristics or sensory quality of the produced wines through the synthesis of mycotoxins and off-flavors, respectively [22]. Mycotoxins are secondary metabolites that can be extremely harmful to human and animal health [23]. It has been reported that exposure of consumers to mycotoxins may have carcinogenic, teratogenic, mutagenic, or even immunosuppressive effects [24]. In addition, contamination of vineyards with fungi may lead to important economic losses in the viticulture sector; therefore, it is of major importance that such infections be avoided prior to wine production [25]. Two main strategies are used for control of fungal contamination, namely physical and chemical strategies. Physical strategies refer to the breeding for *Fusarium*-resistant plants, while chemical strategies refer to pre-harvest use of fungicides [26]. The most commonly used strategies against grape fungal infections to date involve the application of chemical and biological fungicides [25]. Various fungicides are commercially available nowadays and can be applied to vineyards in accordance with good agricultural practices (GAP). Although fungicides can be applied in accordance with GAP, fungicide residues may still be present in the grape juice, negatively influencing wine qualities such as color and aromatic and phenolic profiles [27,28,29,30,31]. Myclobutanil is a chiral triazole fungicide with the chemical name 2-*p*-chlorophenyl-2-(1H-1,2,4,-triazole-1-ylmethyl)-hexanenitrile, and is mostly employed to control various fungal diseases that may occur in grapes, cereals, and fruits and vegetables in general [32]. More specifically, when myclobutanil is applied to grapes it acts against fungi of the taxa Ascomycetes, Deuteromycetes, and Basidiomycetes, which cause various diseases during grape growth [32]. Although myclobutanil’s acute toxicity is low—its half life in grape foliage has been estimated at approximately 15 days—it has been reported recently that myclobutanil cannot be metabolized by human enzymes. In addition, when myclobutanil is consumed, it may cause various toxic health effects, such as hepatocyte hypertrophy and testicular atrophy in rodents [33,34]. It has also been reported to increase liver mixed function oxidase and to disrupt steroid hormone homeostasis [35,36,37,38]. Consequently, it is important to eliminate any and all myclobutanil fungicide from produced wines.

Within this context, two newly isolated yeast strains from grapes were examined regarding their physiological behavior regarding sufficient alcohol production [39] in non-previously-sterilized fermentation media composed of grape must, in which very high initial sugar concentrations were employed, and media were supplemented with myclobutanil. The ability to remove myclobutanil pesticide during fermentation simultaneously with significant ethanol production was monitored and has been critically considered and discussed.

## 2. Materials and Methods

### 2.1. Yeast Isolation and Molecular Identification

Two *Saccharomyces cerevisiae* strains from the culture collection of the Laboratory of Microbiology and Biotechnology of Foods (Department of Food Science and Human Nutrition, Agricultural University of Athens, Athens, Greece), isolated from grapes, were used in this study: LMBF-Y 16 and LMBF-Y 18. The two strains were initially revived from stock cultures (−80 °C) by inoculation of 100 μL in 10 mL of yeast extract peptone dextrose (YPD) medium containing 1% (*w*/*v*) yeast extract (Difco Laboratories, Detroit, Michigan, MI, USA), 2% (*w*/*v*) Bacto peptone (Difco), and 1% (*w*/*v*) glucose. The cultures were incubated at T = 27 °C for 24 h and then streaked on YPD 1% (*w*/*w*) agar plates. A single colony from each plate was transferred to 10 mL of YPD medium and, after 24h of incubation at *T* = 27 °C, DNA extraction was realized [40]. Genomic DNA was amplified by PCR reaction targeting the D1/D2 region of 26S rRNA gene using the primers NL-1 (5-GCATATCAATAAGCGGAGGAAAAG-3) and NL-4 (5-GGTCCGTGTTTCAAGACGG-3) [41]. PCR products were visualized on agarose 1% (*w*/*w*) gel, purified with the QIAquick^®^ PCR Purification Kit (Qiagen, Hilden, Germany) according to the manufacturer’s instructions and sequenced. Identification queries were performed using the BLASTN program as well as the YeastIP databases [42]. The two isolates were characterized at strain level by the technique developed by Legras et al. [43,44], based on the optimized interdelta primers (delta 12-delta 21).

### 2.2. Yeast Strains and Culture Conditions

*S. cerevisiae* LMBF-Y 16 and *S. cerevisiae* LMBF-Y 18 strains were regenerated in YPDA slants (20 g/L glucose, 10 g/L yeast extract, 10 g/L peptone, and 25 g/L agar) every 4 months in order to maintain yeast viability. Pre-cultures were performed in 250 mL non-baffled conical flasks filled with 50 mL of medium (YPD medium: 20 g/L glucose, 10 g/L yeast extract, 10 g/L peptone, pH ≈ 3.5) previously autoclaved at T = 115 °C at 1.5 atm for 15 min. Trials were performed in 250 mL (filled with 50 mL medium) and 2.0 L (filled with 1.5 L medium) agitated flasks filled with either grape must, to which commercial sugars were added (glucose and fructose provided by the Hellenic Industry of Sugar SA, Orestiada, Greece) or with glucose-based synthetic media. Initial total sugar (TS_0_) concentrations in the media were adjusted to approximately 150, 220, and 240 g/L. The TS_0_ concentration in the grape musts was approximately 150 g/L; therefore, if trials with higher TS_0_ were carried out, equimolar fructose and glucose quantities were added into the medium. All grape-must-based media were enriched with 0.3 g/L of (NH_4_)_2_SO_4_, while medium pH value was adjusted to 3.5 ± 0.2. Synthetic glucose-based media presented the following salt composition in g/L: KH_2_PO_4_ 7.0, Na_2_HPO_4_ 2.5, MgSO_4_∙7H_2_O 1.5, CaCl_2_∙2H_2_O 0.15, FeCl_3_∙6H_2_O 0.15, ZnSO_4_∙7H_2_O 0.02, and MnSO_4_∙H_2_O 0.06 [45]. The nitrogen sources used were ammonium sulfate and yeast extract (concentrations 7.0 and 1.0 g/L, respectively), while initial glucose was added to approximately 220 g/L. The pH value was adjusted to 3.5 ± 0.2.

Non-previously-sterilized media were used as the substrate of each fermentation batch. Before inoculation, conical flasks were subjected to heat treatment (pasteurization, 10 min, T = 95 °C). Immediately after cooling the flasks, inoculation with exponential pre-culture (pre-culture carried out for approximately 24 h in YPD medium; see previous) was performed. For the 250 mL flask trials, 1 mL of pre-culture was aseptically inoculated into every flask, previously filled with 50 mL of medium (thus, a 2% *v*/*v* inoculation occurred). For the 2.0 L flasks, 50 mL of exponential pre-culture was added into 1450 mL of the medium (thus, a 3.3% *v/v* inoculation occurred). All flasks were incubated aerobically in an orbital shaker (New Brunswick Scientific, Enfield, CT, USA) with an agitation rate of 180 rpm at T = 25 ± 1 °C [46].

The possibility of removal of myclobutanil fungicide from the growth medium was also investigated. Myclobutanil was added to all non-control media at concentrations of 0.1 mg/L and 1.0 mg/L. Successive batch fermentations were carried out under the same aerated conditions (agitated flasks).

### 2.3. Analytical Methods

The whole content of the 250 mL flasks (50 ± 1 mL) or samples from the 2.0 L flask (approximately 20 mL) were collected and yeast biomass was harvested by centrifugation at 9055 *g* for 10 min at T = 21 ± 1 °C (Suprafuge, Heraeus Sepatech, Midland, ON, Canada), washed twice with distilled water, and centrifuged again. Yeast cell concentration was determined gravimetrically by placement of wet biomass at T ≈ 105 °C until constant weight and was expressed as dry cell weight (DCW) (X, g/L). The pH values were measured using a digital pH meter (Jenway 3020, Cole-Parmer, Staffordshire, UK). The reducing sugar concentration was determined according to the dinitrosalicylic acid (DNS) method, which gives a rapid, simple and accurate estimation of the reducing sugar concentration in various media [47], measured at 540 nm (Spectrophotometer, U-200, Hitachi, Fukuoka, Japan) and expressed as glucose equivalent.

Ethanol, glycerol, and (in several trials) glucose and fructose were quantified through high-performance liquid chromatography (HPLC) in a Waters Association 600E apparatus equipped with a RI detector (Waters 410, Midland, ON, Canada). An ion exclusion column (300 nm × 7.8 mm) Aminex HPX-87*H* (Bio-Rad, CA, USA) was used for separation of the compounds. The mobile phase was H_2_SO_4_ 0.005 mol/L. The column temperature was set at T = 65 °C with a flow rate of 0.8 mL/min [48]. The injection volume was 20 μL. For quantitative analysis, standard solutions of ethanol and glycerol (Sigma-Aldrich Ltd., Taufkirchen, Germany) were prepared in pure water (Milli-Q, Merk, Taufkirchen, Germany) at various concentrations. For all trials, all experimental points presented in the tables and figures are the mean values of two determinations.

In the 250 mL shake-flasks, dissolved oxygen tension (DOT, in % *v*/*v*) was determined off Line using a selective electrode (OXI 96, B-SET, Weilheim, Germany) [49]. Before harvesting, the shaker was stopped and the probe was placed into the flask, after which the shaker was again switched on and the measurement was taken after DOT equilibration (this usually happened within the next 10 min). For all culture phases tested in the 250 mL trial, DOT values were always ≥25% *v*/*v*.

Concentration of myclobutanil (C_15_H_17_ClN_4_) in the fermented medium was determined by a GC-electron capture detector (ECD, Shimadzu, Kyoto, Japan) [50]. The GC-ECD analysis was performed on a GC-17A chromatograph (Shimadzu AOC-zoi auto injector, Shimadzu, Kyoto, Japan) equipped with an ECD detector (Shimadzu, Kyoto, Japan). The method was based on the extraction of the targeted compound with an organic solvent. In more detail, the extraction was conducted by mixing 10 mL of the sample with 20 mL of acetone and homogenizing for approximately 1 min [51], after which 20 mL of dichloromethane and 20 mL of petroleum ether were added, and the mixture was further homogenized and centrifuged. After centrifugation, 25 mL of the supernatant was placed in a water bath (T ≈ 75 °C) and was heated until dry. The dry residue was then redissolved in 5.0 mL of an isooctane–toluene mixture (9:1, *v*/*v*) and analyzed by GC-ECD.

For quantitative analysis, standard solutions of myclobutanil (water solubility: 142 mg/L) (Sigma Aldrich Inc., Taufkirchen, Germany) were prepared at various concentrations [52]. The stock solutions were stored at low temperature (−18 °C) in glass containers sealed with Teflon lids. The injection volume was 1 μL. A DB-WAX highly polar column (30 m × 0.32 mm) was used for analysis [53]. The GC-ECD operating conditions were set as follows: the temperature program was initially set at T = 40 °C for 5 min and the temperature gradient at T = 30 °C/min to final temperature T = 230 °C for 30 min. Injector temperature was set at T = 210 °C. Detector temperature was set at T = 300 °C. Ethanol absolute and pure water (Milli-Q, Merk) were also used. Carrier gas was helium at constant pressure (10.36 psi) with a nominal flow rate of 1.0 mL/min.

### 2.4. Repeatability

For all trials, all experimental points that are presented throughout the text in both tables and figures are the mean values of two replications (SE < 15%).

## 3. Results and Discussion

### 3.1. Yeast Molecular Identification

**Figure 1:**
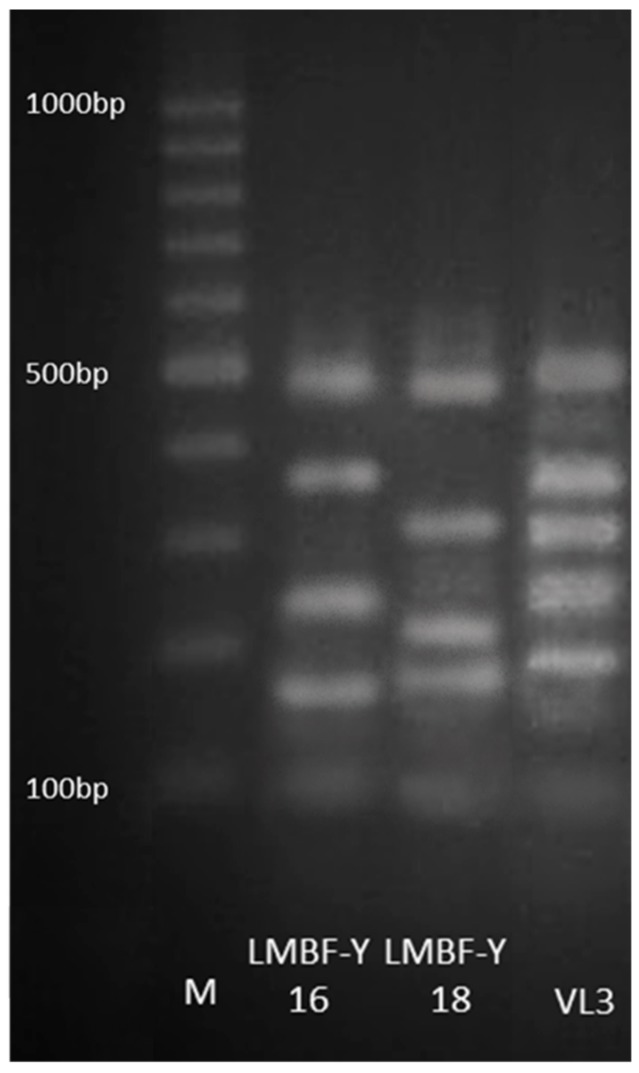
Electrophoretical patterns obtained for the two tested yeast strains, LMBF-Y16 and LMBF-Y18, with delta12–delta21 primers. The commercial *Saccharomyces cerevisiae* strain VL3 (Laffort) was used as a positive control. A 100 bp DNA ladder marker (BioRad) served as the size standard.

The two isolates were identified as *Saccharomyces cerevisiae* based on the sequence data analysis of the D1/D2 region of 26S rRNA gene. In order to confirm that the two isolates of *S. cerevisiae* corresponded to two different strains, amplification of the interdelta region was performed by using the primers delta 12-delta 21 [43]. The obtained electrophoretical patterns for LMBF-Y16 and LMBF-Y18 were different, confirming the existence of the two different *S. cerevisiae* strains that were used in the present study. Additionally, the commercial strain VL3 (Laffort) was used as a positive yeast control species for the inter delta PCR reaction (Figure 1).

### 3.2. Medium and Shake-Flask Culture Optimization

In the second part of the study, optimization of the medium composition and the shake-flask culture configuration was carried out. In this part of the experiment, the microorganism *S. cerevisiae* LMBF-Y 16 was employed, and comparisons between the utilization of the salt-supplemented glucose-based medium with the medium composed of sugar-enriched grape must, as well as between the trials performed in 2.0 L and 250 mL flasks were carried out. The obtained results are depicted in Table 1.

In order to demonstrate the repeatability of the cultures, in one experiment (*S. cerevisiae* LMBF-Y 16 cultivated in 250 mL flask experiments in salt-supplemented glucose-based media) and at one experimental point (at fermentation time t = 74 h after inoculation), seven flasks were simultaneously removed from the incubator. The obtained result was as follows: for yeast DCW (X, g/L), minimum and maximum values were 5.10 and 6.20 g/L, with a mean value of 5.68 g/L, an obtained standard deviation of 0.481, a standard error of 0.182, and a variance of 0.231. For the case of the remaining non-consumed total sugars (TS_r_, g/L), maximum and minimum values were 90 and 105 g/L respectively, with a mean value of 96.17 g/L, an obtained standard deviation of 6.146, a standard error of 2.322, and a variance of 37.772. For ethanol (Eth, g/L), minimum and maximum values were 45.1 and 48.0 g/L, respectively, with a mean value of 46.54 g/L, an obtained standard deviation of 0.947, a standard error of 0.358, and a variance of 0.896. For glycerol (Gly, g/L), minimum and maximum values were 3.30 and 4.40 g/L, with a mean value of 3.77 g/L, an obtained standard deviation of 0.341, a standard error of 0.129, and a variance of 0.116. Finally, for the dissolved oxygen tension (DOT, % *v*/*v*), minimum and maximum values were 24% and 32% *v*/*v*, with a mean value of 28.11%, an obtained standard deviation of 2.970, a standard error of 1.122, and a variance of 8.821.

**Figure 2:**
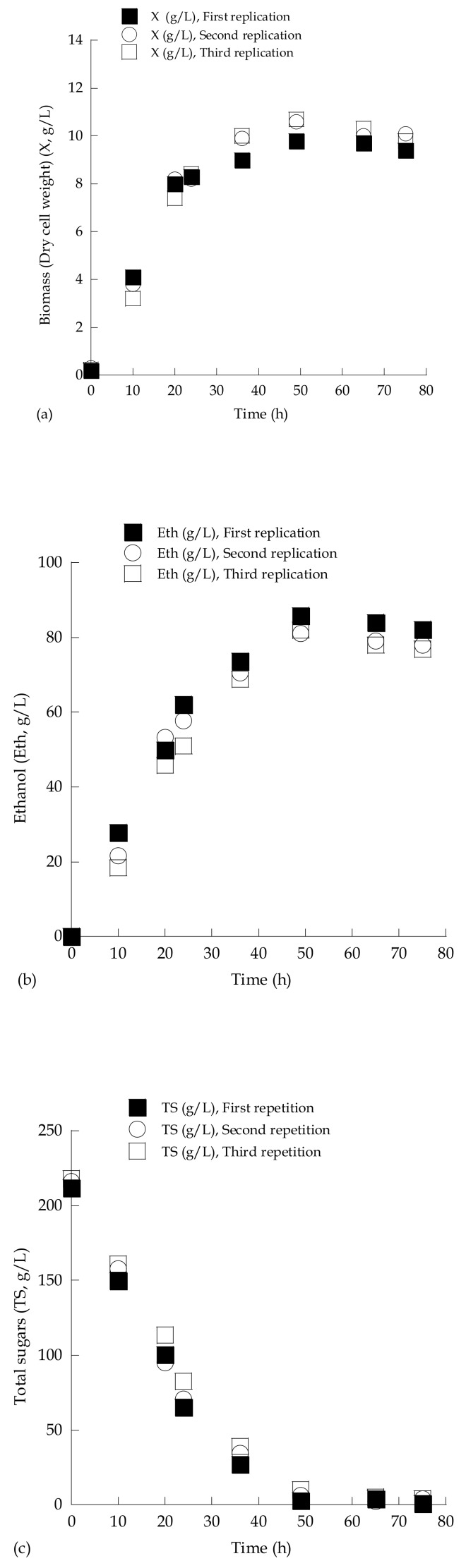
Kinetics of biomass (yeast dry cell weight, DCW) (X, g/L) (**a**), ethanol (Eth, g/L) (**b**), and total sugars (TS, g/L) (**c**) during growth of *Saccharomyces cerevisiae* strain LMBF-Y 16 on sugar-enriched grape must with initial total sugar concentration (TS_0_) adjusted to *c.* 220 g/L. Culture conditions: growth on 2.0 L flasks previously pasteurized (10 min, T = 95 °C), at 180 ± 5 rpm, pH value throughout the culture = 3.5 ± 0.2, incubation temperature T = 25 ± 1 °C. Three replications of the same experiment are presented. Each experimental point presented in the runs is the mean value of two independent measurements (SE < 15%).

To further demonstrate the repeatability of the performed trials, one experiment (*S. cerevisiae* LMBF-Y 16 growing on media composed of sugar-enriched grape must with initial total sugar concentration adjusted to approximately 220 g/L; Figure 2) was repeated three consecutive times, and the results concerning biomass (DCW) production, ethanol biosynthesis, and consumption of total sugars, illustrated in Figure 2, demonstrated satisfactory repeatability.

**Figure 3:**
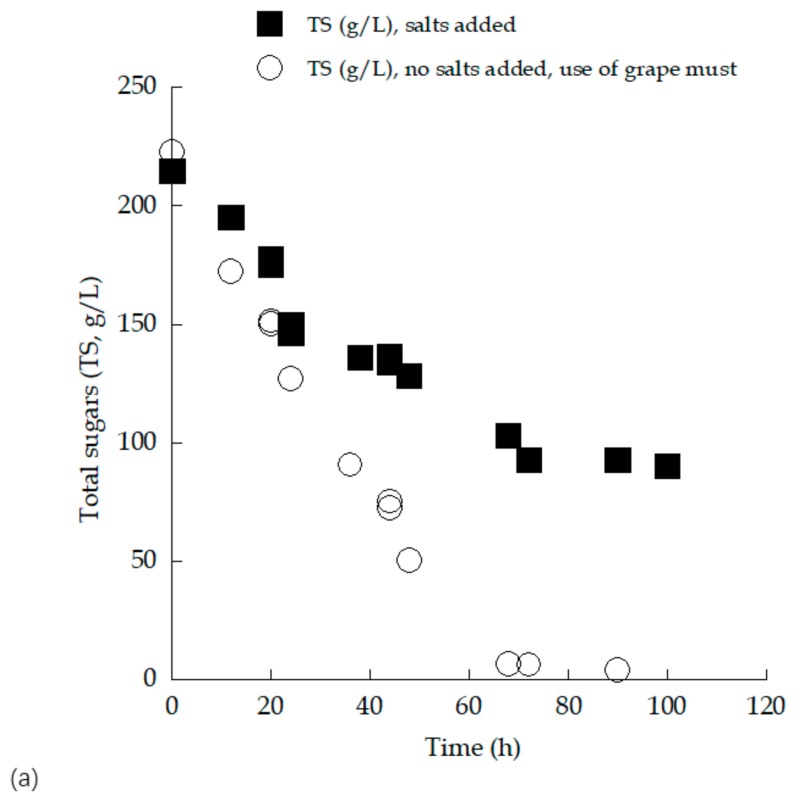

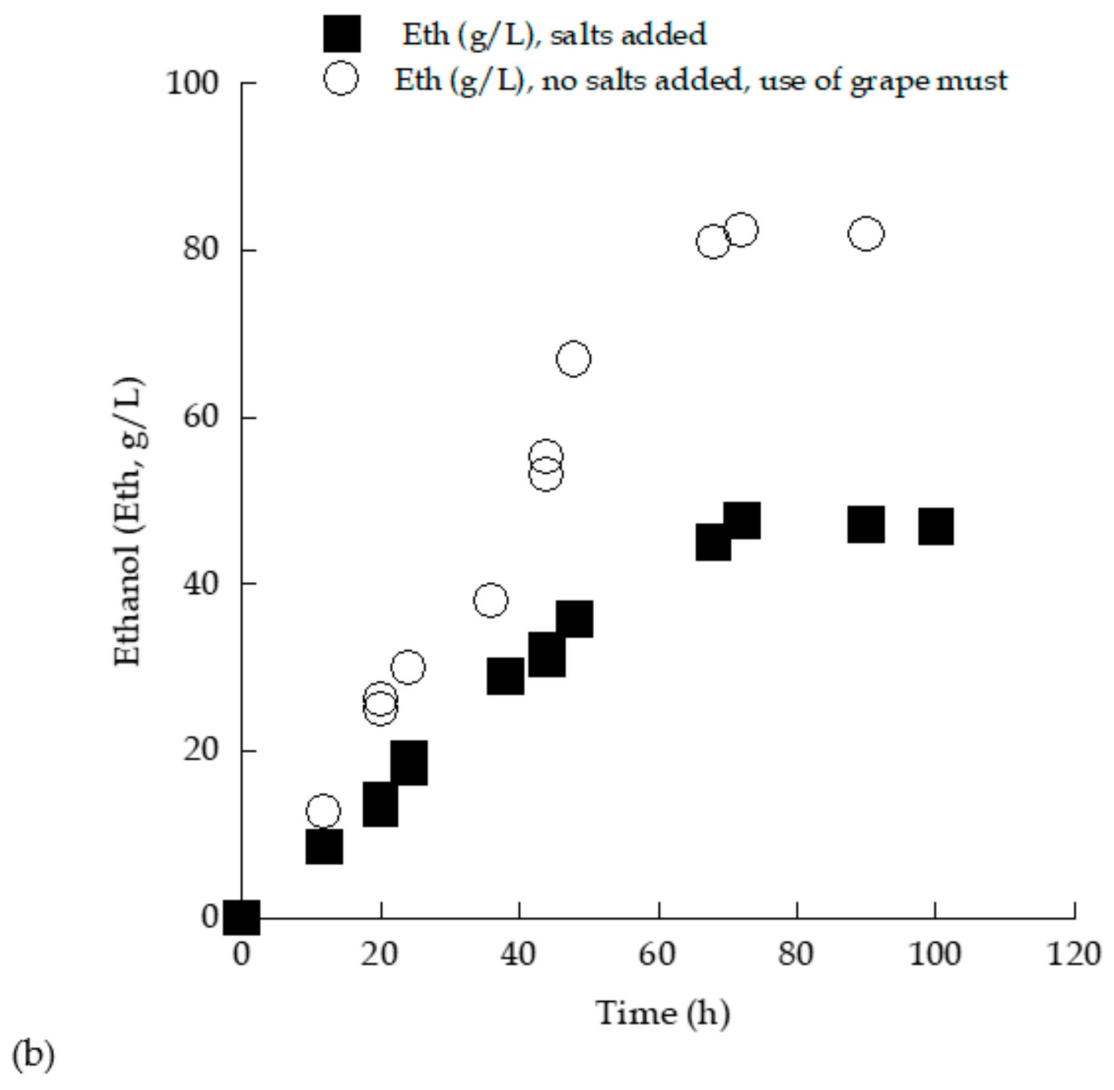
Evolution of total sugars (TS, g/L) (**a**) and ethanol (Eth, g/L) (**b**) during growth of *Saccharomyces cerevisiae* strain LMBF-Y 16 on either glucose-based salt-enriched synthetic media or sugar-enriched grape must with initial total sugar concentration (TS_0_) ≈ 220 g/L. Culture conditions: growth on 250 mL flasks previously pasteurized (10 min, T = 95 °C) at 180 ± 5 rpm, pH value throughout the culture = 3.5 ± 0.2, incubation temperature T = 25 ± 1 °C. Each point is the mean value of two independent measurements (SE < 15%). Each point is the mean value of two independent measurements (SE < 15%).

From the kinetic results demonstrated in Table 1, it can be seen that in the trials performed in the (sugar-enriched) grape must, noticeably higher quantities of sugars were assimilated, and significantly higher ethanol quantities were produced compared with the trials performed in the glucose-based synthetic medium. On the other hand, synthesized glycerol quantities in absolute values (g/L) were almost equal in both trials (approximately g/L), although in the synthetic glucose-based culture, noticeably lower sugar quantities had been assimilated compared with the grape must experiment. Comparisons of the kinetics of sugars assimilated and ethanol produced are shown in Figure 3.

From the above-mentioned analysis, as illustrated in Figure 3, it was demonstrated that the addition of salts into the medium did not provide a significant positive effect upon the fermentative efficiency of *S. cerevisiae*. Similarly, the addition of salts into the medium negatively affected both biomass (DCW) and ethanol production by another *S. cerevisiae* strain (MAK-1) performing alcoholic fermentation under aerobic conditions in shake-flask molasses-based, non-aseptic experiments [46].

**Figure 4:**
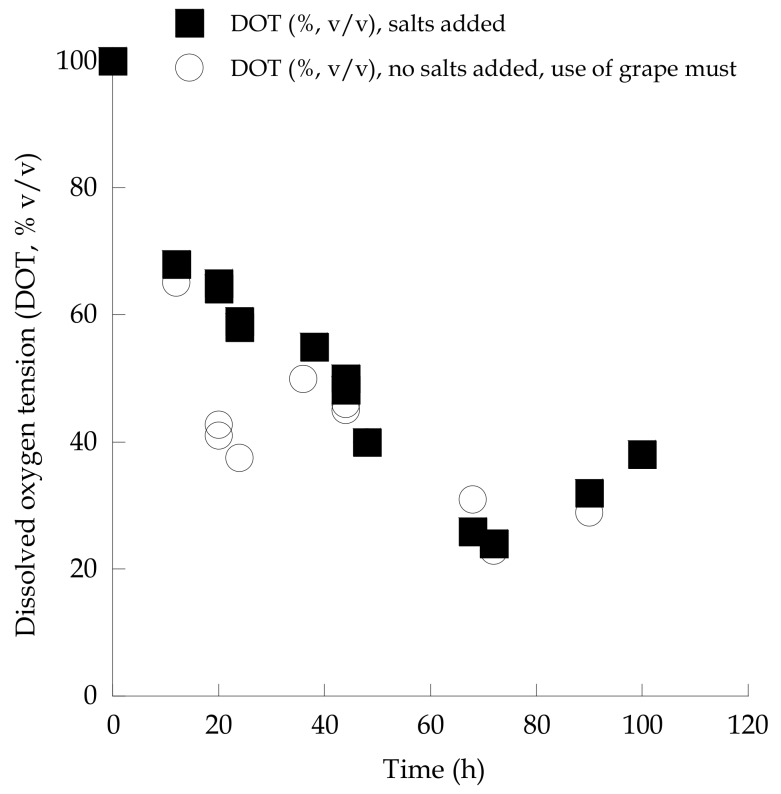
Evolution of dissolved oxygen tension (DOT, % *v*/*v*) during growth of *Saccharomyces cerevisiae* strain LMBF-Y 16 on either glucose-based, salt-enriched synthetic media or sugar-enriched grape must with initial total sugar concentration ≈ 220 g/L. Each point is the mean value of two independent measurements (SE < 15%).

Oxygen saturation into the medium (DOT, % *v*/*v*) was measured in both the 250 mL shake-flask experiments performed, and, in both instances, DOT values for all fermentation points were >25% *v*/*v* (Figure 4). It can thus be assumed, that cultures in such types of batch experiment (trials in 250 mL flasks filled with 50 mL of medium) were carried out under fully aerobic conditions [49,54,55,56]. Moreover, as was anticipated [46,48], significant quantities of ethanol were accumulated into the culture medium, since *S. cerevisiae* is one of the most typical “*Crabtree*-positive” yeast species known to exist in the literature [5,57]. Interestingly, in the trial carried out in the 2.0 L flasks, significantly higher yeast DCW production occurred in comparison with the experiments in 250 mL cultures (X = 9.8 g/L, Y_X/TS_ = 0.047 g/g; the DCW_max_ concentration achieved later was ≈ 11.0 g/L—kinetics not presented), suggesting even higher oxygen saturation in the 2.0 L flask experiments compared with the 250 mL ones. In addition, significantly higher sugar assimilation and somewhat higher ethanol production rates were observed in the 2.0 L shake flasks trials filled with grape must (Table 1), providing evidence that grape musts are very suitable microbial substrates for this type of conversion. Thus, all the subsequent experiments were carried out in grape-must-based media in 2.0 L agitated flasks.

### 3.3. Trials in 2.0 L Flasks with (Enriched) Grape Must Employed as Substrate—Effect of Myclobutanil Addition on Cell Growth

In the third part of this study, trials employing both strains (LMBF-Y16 and LMBF-Y18) were carried out in 2.0 L shake-flasks using media enriched with equimolar quantities of commercial glucose and fructose, under aerobic conditions. Myclobutanil addition also took place at initial concentrations of 0.1 and 1.0 mg/L. Cultures were carried out at TS_0_ quantities adjusted to ~150 and ~250 g/L (in the latter case, very-high-gravity fermentations were conducted), and the kinetic behavior of the strains is presented in Table 2 and Table 3. According to the results obtained, both *S. cerevisiae* LMBF-Y 16 and LMBF-Y 18 strains were characterized by high fermentation capacity, as they consumed all available sugars of the media while simultaneously showing high biomass production. Significant amounts of biomass (yeast DCW) at concentrations ranging from 7.6 to 10.6 g/L were recorded for both yeast strains regardless of the presence of myclobutanil fungicide in the growth medium. More specifically, the biomass of *S. cerevisiae* LMBF-Y 16 produced in the fermentation medium where initial sugar concentration was 150 g/L reached maximum values of 9.1 and 9.3 g/L for the control and the enriched sample with the lowest fungicide concentration (0.1 mg/L myclobutanil), respectively. Finally, in the case where the substrate was enriched with 1.0 mg/L of myclobutanil, biomass was slightly reduced (8.4 g/L). In the case where the TS_0_ concentration was adjusted to approximately 250 g/L, biomass production of *S. cerevisiae* LMBF-Y 16 was enhanced by the presence of the fungicide (Table 2b). A similar observation was also made for *S. cerevisiae* LMBF-Y 18 (Table 3b).

**Figure 5:**
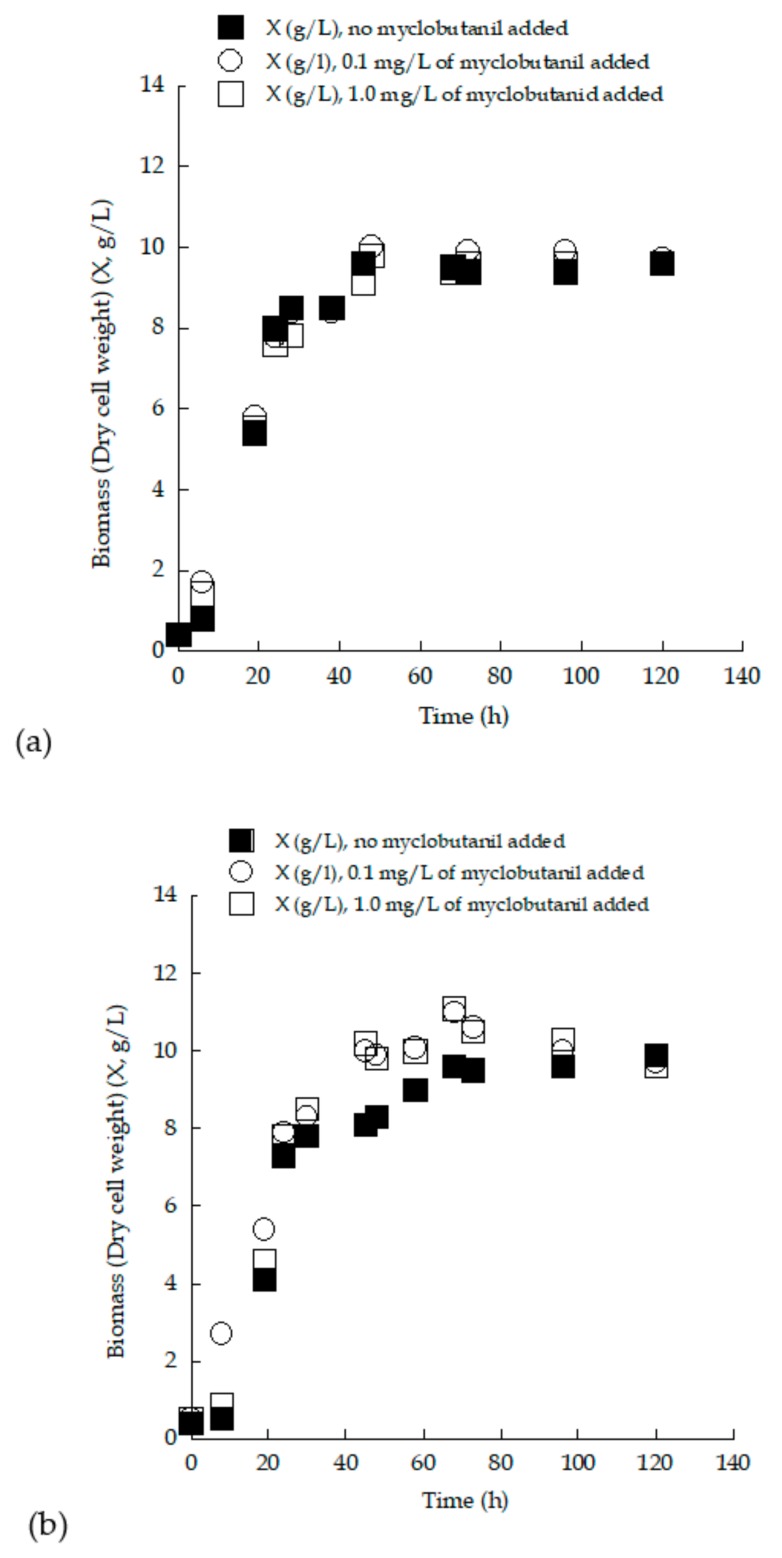
Biomass (yeast dry cell weight) (X, g/L) evolution during growth of *Saccharomyces cerevisiae* strain LMBF-Y 18 on grape-must-based media with initial total sugar concentration (**a**) ~150 g/L and (**b**) ~250 g/L, with or without the addition of myclobutanil fungicide at different concentrations (0.1 mg/L and 1.0 mg/L). Culture conditions as in Table 3. Each point is the mean value of two independent measurements (SE < 15%).

The presence of fungicides was expected to have a negative effect on microorganisms’ growth [58]. However, in the present study, the presence of the myclobutanil fungicide had a favorable effect on the growth of both yeasts. A similar observation was documented by Sarris et al. [30], in which *S. cerevisiae* MAK 1, cultured in shake-flask experiments on sugar-enriched pasteurized grape must, produced biomass at a concentration of approximately 8 g/L, while the addition of quinoxyfen fungicide resulted in yeast biomass content increasing up to a value of 9.5 g/L [30]. Similarly, there have been many documented cases where the addition of constituents that in theory would exert an inhibitory effect increased or maintained the concentration of biomass rather than reducing it [25,59]. To conclude, a thorough investigation targeting the effect of pesticides on each specific strain in order to elucidate the optimum combination is needed for each specific application. The kinetics of yeast DCW production vs. the culture time for the strain *S. cerevisiae* LMBF-Y 18 growing on (enriched) grape musts supplemented with 0.0 (control experiment), 0.1 and 1.0 mg/L of myclobutanil (Figure 5) demonstrated that the added biocide did not result in alteration of cell growth by the strain. Interestingly, at higher TS_0_ concentrations, the addition of myclobutanil seemed to stimulate yeast biomass production (Figure 5b).

**Figure 6:**
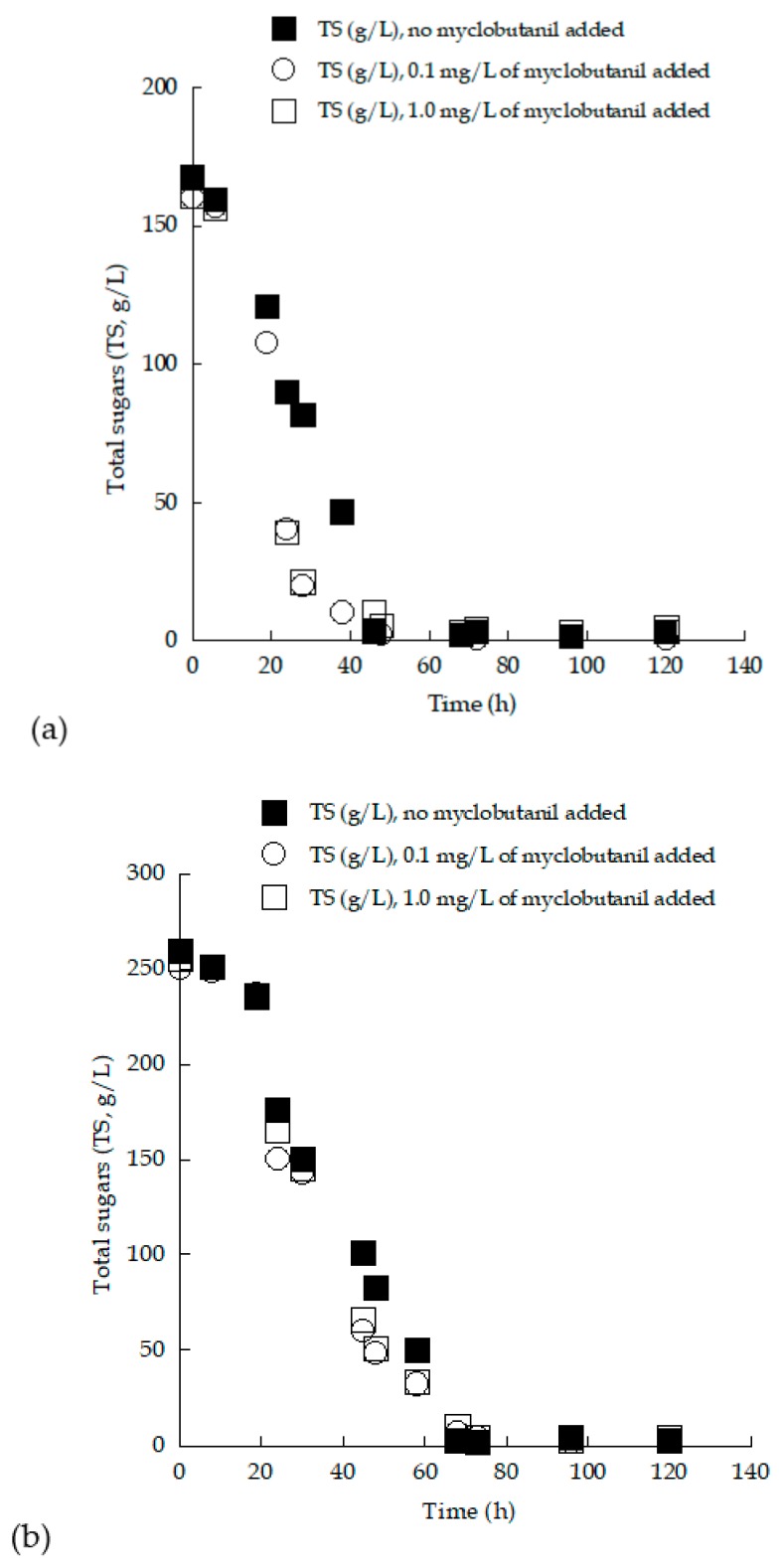
Total sugars (TS, g/L) evolution during growth of *Saccharomyces cerevisiae* strain LMBF-Y 18 on grape-must-based media with initial total sugar concentration (**a**) ~150 g/L and (**b**) ~250 g/L, with or without the addition of myclobutanil fungicide at different concentrations (0.1 mg/L and 1.0 mg/L). Culture conditions as in Table 3. Each point is the mean value of two independent measurements (SE < 15%).

Enriched grape must employed as a substrate contained almost equivalent initial concentrations of glucose and fructose, while in the trials performed with TS_0_ adjusted to approximately 250 g/L, excessively high substrate concentrations were indeed employed (“very-high-gravity fermentation” conditions; [13]). It is well known that *S. cerevisiae* strains can ferment carbon sources like glucose and fructose even in the presence of oxygen, and, in several cases, indeed very high quantities of sugars have been assimilated by the employed strains in batch and/or fed-batch strategies [5,8,12,46,60]. The two newly isolated LMBF-Y 16 and LMBF-Y 18 strains were used in successive batch fermentations with grape must as the fermentation medium, and both yeast strains completed the fermentation rapidly as the final sugar concentration (TS_r_) was ≤5.2 g/L (see Table 2 and Table 3). At this concentration value, it is assumed that alcoholic fermentation under actual winemaking conditions has been adequately completed. The kinetics of total assimilated sugars vs. culture time of *S. cerevisiae* LMBF-Y 18 strain growing on (enriched) grape musts supplemented with 0.0, 0.1, and 1.0 mg/L of myclobutanil (Figure 6), were characterized by almost equivalent profiles, providing evidence that the addition of the biocide did not alter the sugar consumption rate of the employed strain under the present culture conditions.

**Figure 7:**
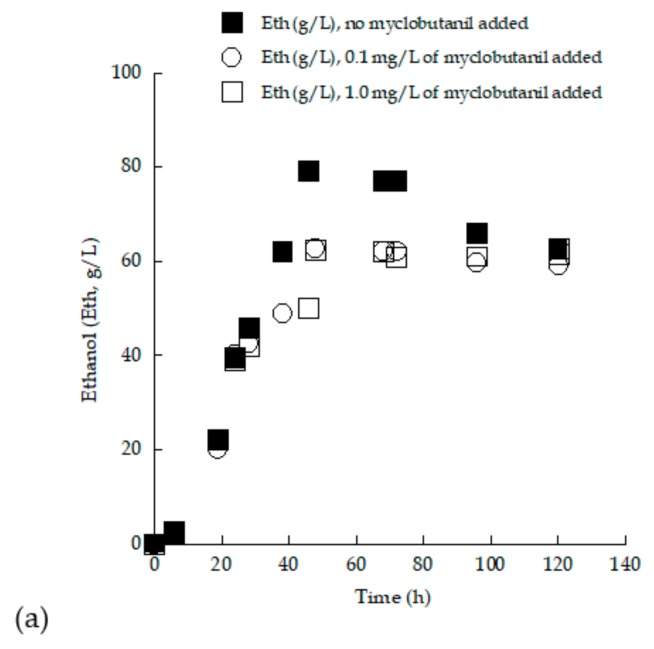

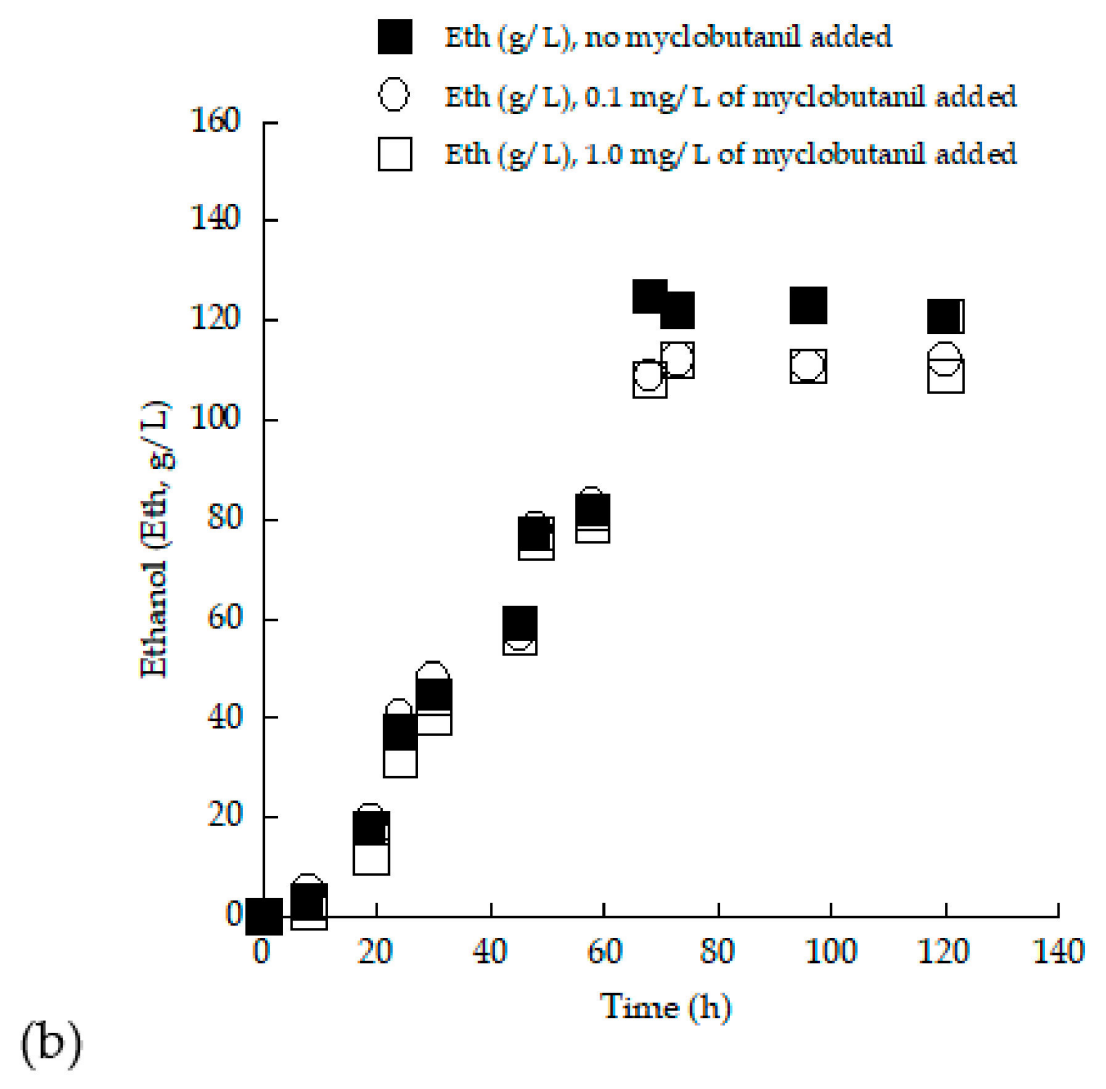
Ethanol (Eth, g/L) evolution during growth of *Saccharomyces cerevisiae* strain LMBF-Y 18 on grape-must-based media with initial total sugar concentration (**a**) ~150 g/L and (**b**) ~250 g/L, with or without the addition of myclobutanil fungicide at different concentrations (0.1 mg/L and 1.0 mg/L). Culture conditions as in Table 3. Each point is the mean value of two independent measurements (SE < 15%).

The alcoholic fermentation process is a crucial step in the production of wine [61]. It is known that yeast strains are able to grow exponentially when sugars are available in the fermentation medium, producing ethanol as a perfectly growth-associated product of the process [5]. In the present study, ethanol production of the two novel yeast strains was monitored during fermentation with TS_0_ concentrations of approximately 150 and 250 g/L. As seen in Table 2 and Table 3, both strains were capable of fermenting sugars and producing ethanol in significant amounts under aerated conditions (*Crabtree* effect), which can be often observed in yeast genera where the microbial metabolism is shifted toward the synthesis of ethanol in spite of the significant oxygen quantities present in the culture medium [46,60]. As it can be noticed from the kinetic results obtained, the addition of biocide somehow negatively affected the maximum quantity of ethanol achieved for both employed strains. Specifically, for the case of the strain LMBF-Y 18, the addition of myclobutanil noticeably reduced the maximum quantity of ethanol (Eth_max_) for both the trials with TS_0_ adjusted to approximately 150 and 250 g/L (Figure 7). In the former case, the blank experiment resulted in an Eth_max_ quantity of 79.0 g/L, with the respective product concentration being reduced to 60.1 g/L upon biocide addition. However, in the case of the blank experiment at TS_0_ ≈ 250 g/L, Eth_max_ concentration was reduced from approximately 125.0 g/L to 112 g/L with the addition of myclobutanil into the medium. The kinetics of ethanol synthesized vs. the culture time for the strain *S. cerevisiae* LMBF-Y 18 growing on (enriched) grape musts supplemented with 0.0 (control experiment), 0.1, and 1.0 mg/L of myclobutanil can be seen in Figure 7, demonstrating the negative effect of the addition of the biocide upon the production of ethanol.

The maximum ethanol yield per unit of sugar consumed (Y_Eth/TSmax_) for most classical microbial sources of ethanol fermentation, namely *S. cerevisiae* and *Zymomonas mobilis*, is 0.51 g/g [5,10,11,46]. Therefore, conversions performed by the strain LMBF-Y 18 in grape-must-based media without added myclobutanil (Y_Eth/TS_ = 0.48 and 0.49 g/g; see Table 3) corresponded to approximately 95% of the maximum theoretical yield of ethanol production, being among the highest values reported in the international literature. Likewise, the highest Y_Eth/TS_ values achieved for the strain LMBF-Y 16 in grape-must-based media without added biocide (Y_Eth/TS_ = 0.47 and 0.46 g/g; see Table 2) were satisfactory, corresponding to values ≥ 90% of the maximum theoretical yield of alcoholic fermentation. In all cases, and regardless of the presence or absence of myclobutanil in the culture medium, very high final concentrations of ethanol (i.e., Eth > 110 g/L) were synthesized during the very-high-gravity fermentations performed for both strains (LMBF-Y 16 and LMBF-Y 18). At the same time, the maximum values of the volumetric productivities achieved (ranging between 1.44 and 1.84 g/L/h) were, equally, very satisfactory.

The metrics of ethanol produced in the literature and comparisons with the results achieved in the current submission are illustrated in Table 4. In general, a wide range of substrates have been utilized for ethanol production, including both saccharine and starchy materials. For instance, sweet sorghum juice has been used successfully for ethanol production by *S. cerevisiae* NP01 under high-gravity fermentation, achieving a high yield of productivity (Table 4), while aeration markedly improved ethanol production [12]. Recently, high-lactose-loaded cheese whey (Lactose: 170–190 g/L), was utilized for ethanol fermentation by *Kluyveromyces marxianus* with a mean ethanol production of 83.2 g/L. Among the many studies targeting high ethanol production, the selection of osmotolerant, high ethanol yielding strains has proved essential [11].

It is noticeable that by the end of alcoholic fermentation, specifically in the trials with the lower TS_0_ concentrations, some of the produced ethanol was re-consumed (e.g., Figure 7a). The high ethanol concentration produced after sugar depletion may provoke a change in cell metabolism, switching from a fermentative to a respiratory growth [46,55,56]. As a result, yeast strains consume the ethanol previously accumulated into the growth medium for the production of energy for cell maintenance [46,60,77]. No diauxic growth was observed in the current investigation (see Figure 5 and Figure 7).

Glycerol can be used as a carbon source for various biotechnological applications, with a major advantage of its not exerting the so-called *Crabtree* effect [5,78]. On the other hand, the sugar → glycerol pathway is antagonistic to the sugar → ethanol one. In the present study, both yeast strains were studied regarding glycerol production. As demonstrated in Table 1, Table 2 and Table 3 glycerol production was affected by the initial sugar concentration into the culture medium, the nature of the sugar, and the type of the culture configuration. Specifically, in very-high-gravity fermentations led by *S. cerevisiae* LMBF-Y 16, glycerol biosynthesis was somehow favored, achieving a final value between 5.2–5.4 g/L, whereas in the comparable trials performed with *S. cerevisiae* LMBF-Y 18, lower final glycerol quantities were achieved (see Table 2b and Table 3b). Therefore, it is not surprising that the strain LMBF-Y 18 achieved somewhat higher Eth_max_ quantities compared with the strain LMBF-Y 16. In trials performed with lower TS_0_ concentrations employed for both strains, although slightly lower glycerol absolute values were recorded, in relative values (g of glycerol per g of sugars consumed), glycerol biosynthesis was favored. As has been documented, a number of yeasts can produce glycerol as their main metabolic compound, to the detriment of the synthesis of ethanol, and some yeast strains can utilize glycerol as a carbon source [79]. Finally, the presence of myclobutanil fungicide exerted no significant effect on glycerol production, as demonstrated in Table 2 and Table 3.

### 3.4. Removal of Myclobutanil Pesticide

The possibility of removal of myclobutanil fungicide during alcoholic fermentation by the action of LMBF-Y 16 and LMBF-Y 18 *S. cerevisiae* strains was investigated (Table 5). More specifically, the residual fungicide was determined for each fermentation batch by calculating the rate of removal of myclobutanil from the fermentation medium.

Enhanced myclobutanil removal was observed with the strain LMBF-Y 16 compared to the strain LMBF-Y 18, as shown in Table 5a,b. More specifically, *S. cerevisiae* LMBF-Y 16 achieved a range of 5%–16% *w*/*w* of myclobutanil removal when the fungicide was added at a higher concentration (i.e., 1.0 mg/L), and an enhanced rate ranging between 23%–27% w/w was noted in the case where the fungicide was added at lower concentration (0.1 mg/L). Likewise, *S. cerevisiae* LMBF-Y 18 removed myclobutanil fungicide by 6%–9% w/w when it was added at a higher concentration (1.0 mg/L), and a greater removal was noted, ranging between 16% and 19% *w*/*w*, when presented in the fermentation medium at a lower concentration (0.1 mg/L). In both strains, myclobutanil removal was significantly enhanced by higher sugar concentrations. The literature suggests that myclobutanil fungicide was either decomposed or absorbed by the yeast strains [80]. More precisely, in the second case, cell wall components, especially glucans, can act as toxin adsorbents [81].

## 4. Conclusions

In recent decades, *Saccharomyces cerevisiae* has become a favorite production microorganism, mostly utilized in wine making and industrial biotechnology. In the present study, the two new wild-type *S. cerevisiae* strains isolated from grapes and not previously assessed for their biochemical and kinetic potentialities, namely LMBF-Y 16 and LMBF-Y-18, showed very interesting biochemical and technological characteristics (biomass production, substrate uptake, ethanol biosynthesis), combined with the possibility of myclobutanil fungicide removal during fermentation of grape must enriched with low-cost sugars. Overall, these data allow us to conclude that since both newly isolated *S. cerevisiae* strains were able to produce ethanol in high quantities under non-aseptic aerated conditions, they could be used in the near future by bioethanol industries to achieve a high yields and productivities. Likewise, wine industries might utilize these two strains to achieve complete fermentations while in parallel minimizing any possible health risks associated with the presence of pesticide in grape musts.

## Figures and Tables

**Table 1:** Quantitative data of *Saccharomyces cerevisiae* LMBF-Y 16 growing on salt-supplemented glucose-based synthetic media and media composed of sugar-enriched grape must with initial total sugar concentration of approximately 220 g/L. Representations of biomass (X, g/L), initial total sugar concentration (TS_0,_ g/L), final total sugar concentration (TS_r_, g/L) at the fermentation points when maximum concentration of ethanol (Eth_max_) was achieved. Fermentation time, conversion yield of biomass produced per total sugars consumed (Y_X/TS_, g/g), conversion yield of ethanol produced per total sugars consumed (Y_Eth/TS_, g/g), and glycerol produced (Glol, g/L) are also presented. Culture conditions: growth on 250 mL or 2.0 L flasks previously pasteurized (10 min, T = 95 °C) at 180 ± 5 rpm, pH value throughout the culture = 3.5 ± 0.2, incubation temperature T = 25 ± 1 °C. Each point is the mean value of two independent measurements (SE < 15%).

Medium Type	Time (Hours)	Flask Type	TS_0_ (g/L)	TS_r_ (g/L)	X (g/L)	Eth_max_ (g/L)	Υ_Eth/TS_ (g/g)	Glol (g/L)	Y_X/TS_ (g/g)
Glucose-based, salts added	72	250 mL	214.7	92.5	5.5	47.6	0.39	3.9	0.045
Enriched grape must, no salts	73	250 mL	222.6	6.6	7.9	82.3	0.38	4.1	0.037
Enriched grape must, no salts	49	2.0 L	211.8	2.6	9.8	85.8	0.41	4.2	0.047

**Table 2:** Quantitative data of *Saccharomyces cerevisiae* LMBF-Y 16 grown on grape must with initial sugar concentration (TS_0_) ≈ 150 g/L (**a**) or ≈ 250 g/L (**b**). Representations of biomass (X, g/L), initial sugar (TS_0,_ g/L), final sugar (TS_r_, g/L), and glycerol (Glol, g/L) concentrations at fermentation points when maximum concentration of ethanol (Eth_max_, g/L) was achieved. Fermentation time, conversion yield of biomass produced per total sugars consumed (Y_X/TS_, g/g), and conversion yield of ethanol produced per total sugar consumed (Y_Eth/TS_, g/g) are also presented. Culture conditions: growth on 2.0 L flasks previously pasteurized (10 min, T = 95 °C) at 180 ± 5 rpm, pH value throughout the culture =3.5 ± 0.2, incubation temperature T *=* 25 ± 1 °C. Each point is the mean value of two independent measurements (SE < 15%).

(**a**)								
**Time (h)**	**Myclobutanil (mg/L)**	**TS_0_ (g/L)**	**TS_r_ (g/L)**	**X (g/L)**	**Glol (g/L)**	**Eth_max_ (g/L)**	**Υ_Eth/TS_ (g/g)**	**Y_X/TS_ (g/g)**
45	-	147.8	2.4	9.1	3.8	68	0.47	0.06
46	0.1	150.2	1.7	9.3	4.4	60.1	0.4	0.06
44	1	156.2	5.1	8.4	4.2	62.2	0.41	0.06
(**b**)								
**Time (h)**	**Myclobutanil (mg/L)**	**TS_0_ (g/L)**	**TS_r_ (g/L)**	**X (g/L)**	**Glol (g/L)**	**Eth_max_ (g/L)**	**Υ_Eth/TS_ (g/g)**	**Y_X/TS_ (g/g)**
68	-	243.2	1	7.6	5.4	112.3	0.46	0.03
73	0.1	248.3	3.2	10.2	5.2	105.2	0.43	0.04
69	1	252.2	3	9.5	5.3	105	0.42	0.04

**Table 3:** Quantitative data of *Saccharomyces cerevisiae* LMBF-Y 18 grown on grape must with initial sugar concentration (TS_0_) ≈ 150 g/L (**a**) or ≈ 250 g/L (**b**). Representations of biomass (X, g/L), initial sugar (TS_0,_ g/L), final sugar (TS_r_, g/L), and glycerol (Glol, g/L) concentrations at fermentation points when maximum concentration of ethanol (Eth_max_, g/L) was achieved. Fermentation time, conversion yield of biomass produced per total sugar consumed (Y_X/TS_, g/g), and conversion yield of ethanol produced per total sugar consumed (Y_Eth/TS_, g/g) are also presented. Culture conditions: growth on 2.0 L flasks previously pasteurized (10 min, T = 95 °C) at 180 ± 5 rpm, pH value throughout the culture = 3.5 ± 0.2, incubation temperature T = 25 ± 1 °C. Each point is the mean value of two independent measurements (SE < 15%).

(**a**)								
**Time (h)**	**Myclobutanil (mg/L)**	**TS_0_ (g/L)**	**TS_r_ (g/L)**	**X (g/L)**	**Glol (g/L)**	**Eth_max_ (g/L)**	**Υ_Eth/TS_ (g/g)**	**Y_X/TS_ (g/g)**
46	-	167.6	3.5	9.6	2.4	79	0.48	0.06
48	0.1	160	2.2	10	2.4	62.6	0.4	0.06
48	1	160.6	5.2	9.8	2.2	62.4	0.4	0.06
(**b**)								
**Time (h)**	**Myclobutanil (mg/L)**	**TS_0_ (g/L)**	**TS_r_ (g/L)**	**X (g/L)**	**Glol (g/L)**	**Eth_max_ (g/L)**	**Υ_Eth/TS_ (g/g)**	**Y_X/TS_ (g/g)**
68	-	259.3	2.5	9.6	3.5	125	0.49	0.04
73	0.1	250	3.8	10.6	3.5	112.2	0.45	0.04
73	1	255.2	4	10.5	3	112	0.45	0.04

**Table 4:** Metrics of ethanol production by *Saccharomyces cerevisiae* strains cultured in several fermentation configurations and carbon sources and comparison with the present investigation.

Yeast Strain	Carbon Source	Initial Sugar Concentration (g/L)	EtOH (g/L)	Reference
Bakers’ yeast	Carob pod	200–350	~62	[62]
Bakers’ yeast	Molasses	150–300	53.0	[63]
*S. cerevisiae*	Carob pod extract (Saccharose)	200	95	[64]
*S. cerevisiae* NP01	Sweet sorghum juice	280–300	134.3	[12]
*S. cerevisiae* BY4741	Sweet sorghum juice concentrated	278.6	113.7	[65]
*S. cerevisiae* NP01	Sucrose	280	95.3	[66]
*S. cerevisiae* 27817	Glucose	50–200	5.1–91.8	[67]
*S. cerevisiae* 2399	Glucose	32	13.7	[68]
*S. cerevisiae* 24860	Glucose	150	48.0	[69]
*S. cerevisiae* CMI237	Sugar	160	70.0	[70]
*K. marxianus*	Lactose	170–190	83.2	[71]
*S. cerevisiae* ATCC 24860	Molasses	2–50	5.0–18.4	[72]
*S. cerevisiae* NCYC 1119	Molasses	100	40.0	[73]
*S. cerevisiae* AXAZ-1	Molasses	~216	71.3	[74]
*S. cerevisiae* ATCC 26602	Flour hydrolysates	150	76	[75]
*S. cerevisiae* sp. & *K. marxianus* blends	Henequen juice & molasses blends	~215	41.2	[76]
*S. cerevisiae* MAK-1	Grape must	250	106.4–119.2	[30]
*S. cerevisiae* MAK-1	Olive-mill wastewater & glucose	115	52.0	[48]
*S. cerevisiae* MAK-1	Olive-mill wastewater & molasses	135	52.4	[46]
*S. cerevisiae* LMBF-Y 16	Grape must	250	112.3	Present study
*S. cerevisiae* LMBF-Y 18	Grape must	250	125.0	Present study

**Table 5:** Myclobutanil removal (%, *w*/*w*) and effect on biochemical characteristics (sugar uptake, ethanol biosynthesis, and biomass production) of *S. cerevisiae* LMBF-Y 16 (**a**) and LMBF-Y 18 (**b**).

(**a**)				
***Saccharomyces cerevisiae* LMBF-Y 16**				
Initial sugars (g/L)	~150	~250	~150	~250
Myclobutanil (mg/L)	1	1	0.1	0.1
Ethanol (g/L)	62.2	105	60.1	105.2
Biomass (g/L)	8.4	9.5	9.3	10.2
Myclobutanil decomposition %	5	16	23	27
(**b**)				
***Saccharomyces cerevisiae* LMBF-Y 18**				
Sugars (g/L)	~150	~250	~150	~250
Myclobutanil (mg/L)	1	1	0.1	0.1
Ethanol (g/L)	62.4	112	62.6	112.2
Biomass (g/L)	9.8	10.5	10	10.6
Myclobutanil decomposition %	6	9	16	19

## References

[1] Straathof A.J.J., Wahl S.A., Benjamin K.R., Takors R., Wierckx N., Noorman H.J. (2019). Grand research challenges for sustainable industrial biotechnology. Trends Biotechnol..

[2] Camarasa C., Chiron H., Daboussi F., Della Valle G., Dumas C., Farines V., Floury J., Gagnaire V., Gorret N., Leonil J. (2018). INRA’s research in industrial biotechnology: For food, chemicals, materials and fuels. Innov. Food Sci. Emerg. Technol..

[3] Mohd Azhar S.H., Abdulla R., Jambo S.A., Marbawi H., Gansau J.A., Mohd Faik A.A., Rodrigues K.F. (2017). Yeasts in sustainable bioethanol production: A review. Biochem. Biophys. Rep..

[4] Koutinas A.A., Wang R.H., Webb C. (2007). The biochemurgist -Bioconversion of agricultural raw materials for chemical production. Biofuels Bioprod. Biorefining.

[5] Sarris D., Papanikolaou S. (2016). Biotechnological production of ethanol: Biochemistry, processes and technologies. Eng. Life Sci..

[6] Sonderegger M., Schümperli M., Sauer U. (2004). Metabolic engineering of a phosphoketolase pathway for pentose catabolism in *Saccharomyces cerevisiae*. Appl. Environ. Microbiol..

[7] Widiastuti H., Lee D.-Y., Karimi I.A., Bogle I.D.L., Fairweather M. (2012). In silico analysis to explore the effect of various carbon sources on ethanol production in *Zymomonas mobilis*. Computer Aided Chemical Engineering.

[8] Koutinas A.A., Vlysidis A., Pleissner D., Kopsahelis N., Lopez Garcia I., Kookos I.K., Papanikolaou S., Kwan T.H., Lin C.S.K. (2014). Valorization of industrial waste and by-product streams via fermentation for the production of chemicals and biopolymers. Chem. Soc. Rev..

[9] Selim K.A., El-Ghwas D.E., Easa S.M., Abdelwahab Hassan M.I. (2018). Bioethanol a microbial biofuel metabolite; New insights of yeasts Metabolic Engineering. Fermentation.

[10] Lin Y., Tanaka S. (2006). Ethanol fermentation from biomass resources: Current state and prospects. Appl. Microbiol. Biotechnol..

[11] Puligundla P., Smogrovicova D., Mok C., Obulam V.S.R. (2019). A review of recent advances in high gravity ethanol fermentation. Renew. Energy.

[12] Phukoetphim N., Chan-u-tit P., Laopaiboon P., Laopaiboon L. (2019). Improvement of bioethanol production from sweet sorghum juice under very high gravity fermentation: Effect of nitrogen, osmoprotectant, and aeration. Energies.

[13] Puligundla P., Poludasu R.M., Rai J.K., Reddy Obulam V.S. (2011). Repeated batch ethanolic fermentation of very high gravity medium by immobilized *Saccharomyces cerevisiae*. Ann. Microbiol..

[14] Johnson E.A., Echavarri-Erasun C., Kurtzman C.P., Fell J.W., Boekhout T. (2011). Chapter 3—Yeast Biotechnology. The Yeasts.

[15] Kunkee R.E. (1984). Selection and modification of yeasts and lactic acid bacteria for wine fermentation. Food Microbiol..

[16] Le Jeune C., Erny C., Demuyter C., Lollier M. (2006). Evolution of the population of *Saccharomyces cerevisiae* from grape to wine in a spontaneous fermentation. Food Microbiol..

[17] Suárez-Lepe J.A., Morata A. (2012). New trends in yeast selection for winemaking. Trends Food Sci. Technol..

[18] Querol A., Pérez-Torrado R., Alonso-del-Real J., Minebois R., Stribny J., Oliveira B.M., Barrio E., Toldrá F. (2018). Chapter Five—New Trends in the uses of yeasts in Oenology. Advances in Food and Nutrition Research.

[19] Ganatsios V., Terpou A., Gialleli A.-I., Kanellaki M., Bekatorou A., Koutinas A.A. (2019). A ready-to-use freeze-dried juice and immobilized yeast mixture for low temperature sour cherry (*Prunus cerasus*) wine making. Food Bioprod. Process..

[20] Cappello M.S., Poltronieri P., Blaiotta G., Zacheo G. (2010). Molecular and physiological characteristics of a grape yeast strain containing atypical genetic material. Int. J. Food Microbiol..

[21] Rai A.K., Pandey A., Sahoo D. (2019). Biotechnological potential of yeasts in functional food industry. Trends Food Sci. Technol..

[22] Rousseaux S., Diguta C.F., Radoï-Matei F., Alexandre H., Guilloux-Bénatier M. (2014). Non-Botrytis grape-rotting fungi responsible for earthy and moldy off-flavors and mycotoxins. Food Microbiol..

[23] IARC (1993). Monographs on the Evaluation of carcinogenic risk to humans. Toxins Derived From Fusarium moniliforme: Fumonisins B1 and B2 and Fusarin C.

[24] Marín S., Cano-Sancho G., Sanchis V., Ramos A.J. (2018). The role of mycotoxins in the human exposome: Application of mycotoxin biomarkers in exposome-health studies. Food Chem. Toxicol..

[25] Dzedze N., Van Breda V., Hart R.S., Van Wyk J. (2019). Wine chemical, sensory, aroma compound and protein analysis of wines produced from chemical and biological fungicide treated Chenin blanc grapes. Food Control.

[26] Ben Taheur F., Kouidhi B., Al Qurashi Y.M.A., Ben Salah-Abbès J., Chaieb K. (2019). Review: Biotechnology of mycotoxins detoxification using microorganisms and enzymes. Toxicon.

[27] Briz-Cid N., Castro-Sobrino L., Rial-Otero R., Cancho-Grande B., Simal-Gándara J. (2018). Fungicide residues affect the sensory properties and flavonoid composition of red wine. J. Food Compos. Anal..

[28] Noguerol-Pato R., Sieiro-Sampedro T., González-Barreiro C., Cancho-Grande B., Simal-Gándara J. (2015). Evaluation of the effect of fenhexamid and mepanipyrim in the volatile composition of Tempranillo and Graciano wines. Food Res. Int..

[29] Noguerol-Pato R., Torrado-Agrasar A., González-Barreiro C., Cancho-Grande B., Simal-Gándara J. (2014). Influence of new generation fungicides on *Saccharomyces cerevisiae* growth, grape must fermentation and aroma biosynthesis. Food Chem..

[30] Sarris D., Kotseridis Y., Linga M., Galiotou-Panayotou M., Papanikolaou S. (2009). Enhanced ethanol production, volatile compound biosynthesis and fungicide removal during growth of a newly isolated *Saccharomyces cerevisiae* strain on enriched pasteurized grape musts. Eng. Life Sci..

[31] Sieiro-Sampedro T., Figueiredo-González M., González-Barreiro C., Simal-Gandara J., Cancho-Grande B., Rial-Otero R. (2019). Impact of mepanipyrim and tetraconazole in Mencía wines on the biosynthesis of volatile compounds during the winemaking process. Food Chem..

[32] Athanasopoulos P.E., Pappas C.J., Kyriakidis N.V. (2003). Decomposition of myclobutanil and triadimefon in grapes on the vines and during refrigerated storage. Food Chem..

[33] Fonseca F.S., Carrão D.B., de Albuquerque N.C.P., Nardini V., Dias L.G., da Silva R.M., Lopes N.P., de Oliveira A.R.M. (2019). Myclobutanil enantioselective risk assessment in humans through in vitro CYP450 reactions: Metabolism and inhibition studies. Food Chem. Toxicol..

[34] Ling C., Liew Z., Von Ehrenstein O.S., Heck J.E., Park A.S., Cui X., Cockburn M., Wu J., Ritz B. (2018). Prenatal exposure to ambient pesticides and preterm birth and term low birthweight in agricultural regions of California. Toxics.

[35] Tully D.B., Bao W., Goetz A.K., Blystone C.R., Ren H., Schmid J.E., Strader L.F., Wood C.R., Best D.S., Narotsky M.G. (2006). Gene expression profiling in liver and testis of rats to characterize the toxicity of triazole fungicides. Toxicol. Appl. Pharmacol..

[36] Goetz A.K., Dix D.J. (2009). Mode of action for reproductive and hepatic toxicity inferred from a genomic study of triazole antifungals. Toxicol. Sci..

[37] Goetz A.K., Dix D.J. (2009). Toxicogenomic effects common to triazole antifungals and conserved between rats and humans. Toxicol. Appl. Pharmacol..

[38] Hao W., Zhang Y., Xie Y., Guo B., Chang J., Li J., Xu P., Wang H. (2019). Myclobutanil accumulation, transcriptional alteration, and tissue injury in lizards (*Eremias argus*) treated with myclobutanil enantiomers. Ecotoxicol. Environ. Saf..

[39] Walker G.M., Walker R.S.K. (2018). Enhancing yeast alcoholic fermentations. Advances in Applied Microbiology.

[40] Querol A., Barrio E., Ramón D. (1992). A Comparative study of different methods of yeast strain characterization. Syst. Appl. Microbiol..

[41] Kurtzman C.P., Robnett C.J. (1998). Identification and phylogeny of ascomycetous yeasts from analysis of nuclear large subunit (26S) ribosomal DNA partial sequences. Antonie Van Leeuwenhoek.

[42] Weiss S., Samson F., Navarro D., Casaregola S. (2013). Yeast IP: A database for identification and phylogeny of *Saccharomycotina* yeasts. FEMS Yeast Res..

[43] Legras J.-L., Karst F. (2003). Optimisation of interdelta analysis for *Saccharomyces cerevisiae* strain characterisation. FEMS Microbiol. Lett..

[44] Legras J.L., Merdinoglu D., Cornuet J.M., Karst F. (2007). Bread, beer and wine: *Saccharomyces cerevisiae* diversity reflects human history. Mol. Ecol..

[45] Papanikolaou S., Chevalot I., Komaitis M., Aggelis G., Marc I. (2001). Kinetic profile of the cellular lipid composition in an oleaginous *Yarrowia lipolytica* capable of producing a cocoa-butter substitute from industrial fats. Antonie Van Leeuwenhoek.

[46] Sarris D., Matsakas L., Aggelis G., Koutinas A.A., Papanikolaou S. (2014). Aerated *vs* non-aerated conversions of molasses and olive mill wastewaters blends into bioethanol by *Saccharomyces cerevisiae* under non-aseptic conditions. Ind. Crop. Prod..

[47] Miller G.L. (1959). Use of DNS reagent for the determination of glucose. Anal. Chem..

[48] Sarris D., Giannakis M., Philippoussis A., Komaitis M., Koutinas A.A., Papanikolaou S. (2013). Conversions of olive mill wastewater-based media by *Saccharomyces cerevisiae* through sterile and non-sterile bioprocesses. J. Chem. Technol. Biotechnol..

[49] Palaiogeorgou A.M., Papanikolaou S., de Castro A.M., Freire D.M.G., Kookos I.K., Koutinas A.A. (2019). A newly isolated *Enterobacter* sp. strain produces 2,3-butanediol during its cultivation on low-cost carbohydrate-based substrates. FEMS Microbiol. Lett..

[50] Hrouzková S. (2016). Analytical Methods for Pesticide Detection in Foodstuffs.

[51] Bakırcı G.T., Yaman Acay D.B., Bakırcı F., Ötleş S. (2014). Pesticide residues in fruits and vegetables from the Aegean region, Turkey. Food Chem..

[52] Doulia D.S., Anagnos E.K., Liapis K.S., Klimentzos D.A. (2017). Effect of clarification process on the removal of pesticide residues in white wine. Food Control.

[53] Botezatu A., Pickering G.J., Kotseridis Y. (2014). Development of a rapid method for the quantitative analysis of four methoxypyrazines in white and red wine using multi-dimensional Gas Chromatography—Mass Spectrometry. Food Chem..

[54] Papanikolaou S., Rontou M., Belka A., Athenaki M., Gardeli C., Mallouchos A., Kalantzi O., Koutinas A.A., Kookos I.K., Zeng A.P. (2017). Conversion of biodiesel-derived glycerol into biotechnological products of industrial significance by yeast and fungal strains. Eng. Life Sci..

[55] Piškur J., Rozpędowska E., Polakova S., Merico A., Compagno C. (2006). How did *Saccharomyces* evolve to become a good brewer?. Trends Genet..

[56] Hagman A., Säll T., Compagno C., Piskur J. (2013). Yeast “make-accumulate-consume” life strategy evolved as a multi-step process that predates the whole genome duplication. PLoS ONE.

[57] Rosas-Lemus M., Uribe-Alvarez C., Chiquete-Félix N., Uribe-Carvajal S. (2014). In *Saccharomyces cerevisiae* fructose-1,6-bisphosphate contributes to the Crabtree effect through closure of the mitochondrial unspecific channel. Arch. Biochem. Biophys..

[58] Avenot H.F., Michailides T.J. (2010). Progress in understanding molecular mechanisms and evolution of resistance to succinate dehydrogenase inhibiting (SDHI) fungicides in phytopathogenic fungi. Crop Prot..

[59] Cordero-Bueso G., Arroyo T., Valero E. (2014). A long term field study of the effect of fungicides penconazole and sulfur on yeasts in the vineyard. Int. J. Food Microbiol..

[60] Lavaisse L.M., Hollmann A., Nazareno M.A., Disalvo E.A. (2019). Zeta potential changes of *Saccharomyces cerevisiae* during fermentative and respiratory cycles. Colloids Surf. B Biointerfaces.

[61] Ancín-Azpilicueta C., Jiménez-Moreno N., Sola-Larrañaga C., Galanakis C.M. (2019). Chapter 9–Wine. Innovations in Traditional Foods.

[62] Roukas T. (1994). Continuous ethanol production from carob pod extract by immobilized *Saccharomyces cerevisiae* in a packed-bed reactor. J. Chem. Technol. Biotechnol..

[63] Roukas T. (1996). Ethanol production from non-sterilized beet molasses by free and immobilized *Saccharomyces cerevisiae* cells using fed-batch culture. J. Food Eng..

[64] Sanchez-Segado S., Salar-García M.J., Ortiz-Martínez V.M., de los Ríos A.P., Hernández-Fernández F.J., Lozano-Blanco L.J. (2019). Evaluation of ionic liquids as in situ extraction agents during the alcoholic fermentation of carob pod extracts. Fermentation.

[65] Sasaki K., Tsuge Y., Sasaki D., Kawaguchi H., Sazuka T., Ogino C., Kondo A. (2015). Repeated ethanol production from sweet sorghum juice concentrated by membrane separation. Bioresour. Technol..

[66] Chan-u-tit P., Laopaiboon L., Jaisil P., Laopaiboon P. (2013). High level ethanol production by nitrogen and osmoprotectant supplementation under very high gravity fermentation conditions. Energies.

[67] Vallet C., Saïd R., Rabiller C., Martin M.L. (1996). Natural abundance isotopic fractionation in the fermentation reaction: Influence of the Nature of the Yeast. Bioorganic Chem..

[68] Yu Z., Zhang H. (2003). Ethanol fermentation of acid-hydrolyzed cellulosic pyrolysate with *Saccharomyces cerevisiae*. Bioresour. Technol..

[69] Najafpour G., Younesi H., Syahidah Ku Ismail K. (2004). Ethanol fermentation in an immobilized cell reactor using *Saccharomyces cerevisiae*. Bioresour. Technol..

[70] Navarro A.R., Sepúlveda M.D.C., Rubio M.C. (2000). Bio-concentration of vinasse from the alcoholic fermentation of sugar cane molasses. Waste Manag..

[71] Díez-Antolínez R., Hijosa-Valsero M., Paniagua-García A.I., Gómez X. (2016). Very-high-gravity fermentation of non-supplemented cheese whey permeate by immobilized *Kluyveromyces marxianus*. Chem. Eng. Trans..

[72] Ergun M., Ferda Mutlu S. (2000). Application of a statistical technique to the production of ethanol from sugar beet molasses by *Saccharomyces cerevisiae*. Bioresour. Technol..

[73] Baptista C.M.S.G., Cóias J.M.A., Oliveira A.C.M., Oliveira N.M.C., Rocha J.M.S., Dempsey M.J., Lannigan K.C., Benson P.S. (2006). Natural immobilisation of microorganisms for continuous ethanol production. Enzyme Microb. Technol..

[74] Kopsahelis N., Kanellaki M., Bekatorou A. (2007). Low temperature brewing using cells immobilized on brewer’s spent grains. Food Chem..

[75] Wang R., Ji Y., Melikoglu M., Koutinas A., Webb C. (2007). Optimization of innovative ethanol production from wheat by response surface methodology. Process Saf. Environ. Prot..

[76] Cáceres-Farfán M., Lappe P., Larqué-Saavedra A., Magdub-Méndez A., Barahona-Pérez L. (2008). Ethanol production from henequen (*Agave fourcroydes* Lem.) juice and molasses by a mixture of two yeasts. Bioresour. Technol..

[77] Habegger L., Rodrigues Crespo K., Dabros M. (2018). Preventing overflow metabolism in Crabtree-positive microorganisms through on-line monitoring and control of fed-batch fermentations. Fermentation.

[78] Crabtree H.G. (1929). Observations on the carbohydrate metabolism of tumours. Biochem. J..

[79] Aldiguier A.S., Alfenore S., Cameleyre X., Goma G., Uribelarrea J.L., Guillouet S.E., Molina-Jouve C. (2004). Synergistic temperature and ethanol effect on *Saccharomyces cerevisiae* dynamic behaviour in ethanol bio-fuel production. Bioprocess Biosystesms Eng..

[80] Cabras P., Farris G.A., Fiori M.G., Pusino A. (2003). Interaction between fenhexamid and yeasts during the alcoholic fermentation of *Saccharomyces cerevisiae*. J. Agric. Food Chem..

[81] Piotrowska M., Masek A. (2015). *Saccharomyces Cerevisiae* cell wall components as tools for ochratoxin A decontamination. Toxins.

